# The influence of marketing on the sports betting attitudes and consumption behaviours of young men: implications for harm reduction and prevention strategies

**DOI:** 10.1186/s12954-017-0131-8

**Published:** 2017-01-19

**Authors:** Emily G. Deans, Samantha L. Thomas, Jeffrey Derevensky, Mike Daube

**Affiliations:** 1 Centre for Population Health Research, School of Health and Social Development, Faculty of Health, Deakin University, Melbourne, Victoria Australia; 2 International Centre for Youth Gambling Problems and High Risk Behaviours, McGill University, Montreal, Canada; 3 Faculty of Health Sciences, Curtin University, Perth, Australia

**Keywords:** Marketing, Young men, Sports, Normalisation, Betting, Harm reduction

## Abstract

**Background:**

Gambling can cause significant health and social harms for individuals, their families, and communities. While many studies have explored the individual factors that may lead to and minimise harmful gambling, there is still limited knowledge about the broader range of factors that may contribute to gambling harm. There are significant regulations to prevent the marketing of some forms of gambling but comparatively limited regulations relating to the marketing of newer forms of online gambling such as sports betting. There is a need for better information about how marketing strategies may be shaping betting attitudes and behaviours and the range of policy and regulatory responses that may help to prevent the risky or harmful consumption of these products.

**Methods:**

We conducted qualitative, semi-structured interviews with 50 Australian men (aged 20–37 years) who gambled on sports. We explored their attitudes and opinions regarding sports betting marketing, the embedding of marketing within sports and other non-gambling community environments, and the implications this had for the normalisation of betting.

**Results:**

Our findings indicate that most of the environments in which participants reported seeing or hearing betting advertisements were not in environments specifically designed for betting. Participants described that the saturation of marketing for betting products, including through sports-based commentary and sports programming, normalised betting. Participants described that the inducements offered by the industry were effective marketing strategies in getting themselves and other young men to bet on sports. Inducements were also linked with feelings of greater control over betting outcomes and stimulated some individuals to sign up with more than one betting provider.

**Conclusions:**

This research suggests that marketing plays a strong role in the normalisation of gambling in sports. This has the potential to increase the risks and subsequent harms associated with these products. Legislators must begin to consider the cultural lag between an evolving gambling landscape, which supports sophisticated marketing strategies, and effective policies and practices which aim to reduce and prevent gambling harm.

## Background

Gambling can cause significant health and social harms for individuals, their families, and communities [1, 2]. While academic research has traditionally focused on the harms associated with *problem* or *pathological* levels of gambling, research now suggests that gambling harm may also occur for those with *low* or *moderate* levels of gambling, with the burdens associated with gambling harms now comparable with those associated with alcohol misuse and major depression [3]. Each year, approximately 400,000 Australian adults experience gambling-related harm or are at moderate risk of experiencing harm [4]. The “ripple effect” that gambling can have on families, friends, and employers is also represented by the fact that for each problem gambler, approximately five to ten others are negatively impacted by their gambling [4].

Traditional research paradigms in gambling have predominantly focused on individualised models to explain why some individuals develop problematic or pathological levels of gambling, with personal responsibility approaches offered as key harm minimisation strategies [5]. However, newer research has sought to understand the broader socio-cultural, environmental, and commercial determinants of gambling harm, and the broader range of policy and regulatory strategies that may be used to prevent harm [6–9]. Despite increasing concern from academics, legislators, and community groups about the increasing proliferation of marketing for gambling products and services [10–14], very limited research has explored how marketing strategies may influence gambling attitudes and consumption intentions and the range of strategies that may be used to reduce the risks posed by marketing to different population subgroups.

### Sports betting: the Australian context

Australia arguably has one of the most liberalised and intensive gambling environments in the world [2], with sports betting via online bookmakers a rapidly expanding segment of the Australian gambling market [15, 16]. Official statistics have reported an increase in sports betting expenditure in Australia [17, 18] and increasing profit margins for some online bookmakers [19]. However, this has also coincided with an increase in the number of individuals presenting to clinics for help with problems with this form of gambling, particularly young men [20]. Recent research suggests that approximately three quarters (72.1%) of losses for sports betting come from individuals with some level of gambling problems, representing the greatest proportion of losses derived from people with problem gambling symptoms when comparing across different gambling forms [21]. Further, recent research suggests that there exists a range of factors in both online and land-based environments (including but not limited to accessibility, the role of alcohol, and promotions) that may contribute to risky sports betting behaviours [9].

There has also been a significant increase in the amount of marketing for sports betting products in Australia [22], including significant increases in advertising spend by online bookmakers [23]. Marketing strategies for sports betting extend beyond advertisements on free to air television and also include more contemporary social media platforms such as Twitter and Facebook (which often transcend advertising regulations) [7], as well as commercial sponsorship agreements with sporting codes, stadiums, broadcasters, and individual clubs [10, 24, 25]. For example, there are currently multimillion dollar sponsorship deals between online bookmakers and two of Australia’s major sporting codes—the National Rugby League (NRL) and the Australian Football League (AFL) [26], as well as other codes such as Tennis Australia [27].

### The influence of marketing on sports betting consumption behaviours

Research suggests that young men are the target market for sports betting companies, with a range of marketing and promotional strategies used to both appeal to and reach this key audience segment [8]. Further, some young men have reported they feel targeted and bombarded by sports betting advertising [28]. More broadly, research into the impact of gambling advertising indicates that it may trigger impulses to gamble, may increase already high levels of gambling and may make it more difficult for problem gamblers in particular, to gamble less or not gamble at all [29, 30]. Researchers argue that sports betting has become closely aligned with young men’s sports fan rituals [31], with some researchers highlighting the role that marketing strategies may play in stimulating the risky consumption of sports betting products. For example, researchers have found that sports betting advertising used during sporting matches stimulates a range of positive, negative, and neutral affects in sports betters [32]. Researchers have also demonstrated that specific forms of marketing promotions such as inducements may be particularly influential in stimulating problematic betting behaviours [33].

Marketing research also demonstrates how advertisers may seek to develop upon existing cultural symbols, behaviours, and contexts, with an aim of embedding their product within these behaviours and creating new subcultures and identities associated with that product [34–36]. Researchers have partly documented this process in relation to sports betting advertising. They have analysed the extent to which gambling industry marketing seeks to align sports betting with the culturally valued aspects of being a sports fan—including mateship, support for your team, fan loyalty, thrill, winning, and power [8]. Deans et al. [8] suggest that these marketing tactics, which are so closely aligned with such a valued cultural activity in sports, may have an influential impact on individual and peer group identities associated with gambling on sports. However, few studies have sought to explore how marketing strategies may influence the gambling attitudes and consumption intentions of populations.

## Methods

Utilising in-depth qualitative research with young male sports gamblers (20–37 years), the key target market of betting companies, we aimed to explore the role of marketing in betting behaviours, as well as the range of strategies that may be used to minimise the potential harms associated with marketing. The research was guided by four research questions.

1. How do marketing mechanisms seek to create a cultural alignment between betting and sports?
2. Is there evidence that marketing strategies may be influencing new betting “identities” associated with sports?
3. Do specific forms of promotions encourage young men to gamble more frequently and on events that they would not otherwise bet on?
4. Are there specific strategies that may have the potential to reduce or prevent the risks or harms posed by the marketing for these products?

### Approach

The results presented in this paper were part of a broader study investigating the sports betting attitudes and behaviours of young men [9, 31]. The research used a constructivist grounded theory (CGT) approach [37], acknowledging the active co-creation of knowledge that exists between researchers and research participants. Individuals each have their own socially constructed reality, and the research findings therefore represent a collation of interpretations of multiple lived realities, mutually constructed by the researchers and participants in this study [38]. Such an approach considers (and values) the opinions and experiences of all participants, while attempting to reconstruct their experiences in the most faithful way possible [39].

### Sampling and recruitment strategies

Participants were recruited using purposive [40] and theoretical [41] sampling techniques. We sought to recruit young men who were fans of the National Rugby League (NRL) and/or the Australian Football League (AFL) and who had engaged in betting on these codes. All participants were either current or recent sports bettors, with the exception of one participant who identified as experiencing previous problems with sports betting. While this participant no longer gambled on sports or any other product, the research team chose to include him given his personal experiences with sports betting and to ensure a range of experiences were included in the study sample. We employed a number of strategies to recruit participants including posts on social media (Twitter and Facebook), advertisements on gambling forums, flyers in gyms (and in gym newsletters), and a newspaper article with information about the study. As the study progressed, we relied on snowball techniques to recruit the remainder of participants. Ethical approval was obtained from the University Human Research Ethics Committee. Potential participants were given information about the study and gave their verbal consent prior to completing the interview. Participants received a $30 iTunes, grocery, or petrol voucher for their participation.

### Data collection

Semi-structured, audio-taped interviews lasting between 30 and 60 min were conducted with each participant. The majority of interviews were conducted by author 1, with some conducted by author 2. Demographic information, including age, occupation, highest level of education, and postcode were collected. Participants’ postcodes were used as a guide to assess their socio-economic status, as determined by their Socio-Economic Index for Areas Score (SEIFA) [42]. We used an adapted version of the nine-item Problem Gambling Severity Index (PGSI) developed by Ferris and Wynne, [43] to measure participants’ gambling behaviours, with PGSI scores used to broadly group participants according to their gambling risk characteristics, with a score or 1–2 indicating low levels of problems, 3–7 moderate levels of problems, and 8 and over pathological levels of problems with gambling. However, we also asked more detailed questions about participants’ experiences with gambling and the types of products that they gambled on (including electronic gambling machines (EGMs), greyhound and horse wagering, table games, keno, lotto, and sports betting), as well as the estimated amount spent on a weekly, fortnightly, or monthly basis (depending on the frequency of each participant’s gambling behaviours). Qualitative themes of inquiry included participants’ participation in sports, their early experiences with gambling, and how their gambling behaviours had evolved over time, the role of betting in their peer group rituals related to sports, the changing gambling landscape including the accessibility of online betting products, their attitudes regarding sports betting marketing, and their regulatory suggestions to protect individuals from gambling harm. This paper specifically focuses on participants’ responses to the extent and content of sports betting marketing. Recruitment for the study was stopped when the researchers were confident of a diversity of experiences and opinions to illustrate a number of constructs associated with our research questions and theoretical lens [44].

### Data analysis and interpretation

Data, including participants’ age, occupation, highest level of education, postcode, and SEIFA index, as well as PGSI scores, were entered into SPSS and analysed using basic descriptive statistics. Transcripts were uploaded to QSR NVivo 10 which was used to manage the data. Qualitative data interpretation occurred throughout the interviews and was led by the first two authors who constantly met to discuss the data, and to add new questions to the interview schedule as new ideas, experiences, and opinions emerged from participants’ interviews. Initial data coding, analyses, and interpretation was conducted by authors 1 and 2. We used open-coding techniques to identify narratives that specifically related to the extent and content of sports betting marketing in each of the transcripts. We read and re-read each transcript (and listened to the audio of each interview), employing a constant comparative method [41] to identify the similarities and differences between each interview and to inductively categorise the data [45]. Preliminary findings were discussed amongst the research team for interpretation early on in the data collection/analysis phase. This allowed for emerging themes of inquiry to be identified and guided how best to explore these in subsequent interviews. As noted, we initially explored participants’ attitudes and opinions towards the extent and content of sports betting marketing and then selectively coded the data into distinct subthemes. Attempting to move beyond a descriptive level of analysis, we drew upon relevant literature and theory to help understand and explain further, participants’ experiences.

## Results

### Sample characteristics

The general characteristics of the sample are presented in Table 1. In total, 50 young men participated in the study, aged between 20 and 37 (mean = 28 years, SD = 4). Participants had a range of occupational backgrounds including trade or technician work (*n* = 15), professional work (*n* = 14), community service work (*n* = 7), retail (*n* = 5), management (*n* = 4), labour (*n* = 2), and administrative work (*n* = 2). Just over half (*n* = 29) of the sample had completed an undergraduate or postgraduate University degree, 11 participants had completed secondary schooling, and 10 participants had completed a diploma or advanced diploma at Technical and Further Education (TAFE). More than half of the sample were from moderate (*n* = 24) to high (*n* = 22) socio-economic areas of advantage. PGSI scores ranged from 0 to 11, with over two thirds (*n* = 34) experiencing low or moderate problems with gambling and four receiving a PGSI score indicative of a gambling problem.

**Table 1:** General characteristics

Characteristics	*N* (%)
Age Range	20–37 years (mean 28 years, SD 4)
Occupation	
Technicians and trade workers	15 (30%)
Professionals	14 (28%)
Community and personal service workers	7 (14%)
Sales workers	5 (10%)
Managers	4 (8%)
Labourers	2 (4%)
Clerical and administrative workers	2 (4%)
Unemployed	1 (2%)
Highest level of education	
University	29 (58%)
Secondary	11 (22%)
TAFE	10 (20%)
SEIFA DECILE	
8–10	22 (44%)
4–7	24 (48%)
1–3	4 (8%)
PGSI	
0 (non-problem gambling)	12 (24%)
1–2 (low level of problems)	17 (34%)
3–7 (moderate level of problems)	17 (34%)
8+ (problem gambling)	4 (8%)

Four qualitative themes emerged from the data.

### Prompting and nudging: the role of marketing and promotions in the normalisation of gambling

The first theme to emerge from participants’ narratives related to the changing marketing environment for sports betting products. Table 2 documents the range of marketing channels for sports betting that emerged from participants’ narratives. Most of the marketing strategies for sports betting mentioned by participants were not in environments specifically designed for gambling. Rather, they included television advertising during both regular programming and during sports; at sporting matches; on radio; billboards; public transport, and as pop-ups on social media sites. In gambling environments, participants mentioned that they had seen advertising on mobile sports betting apps, and within clubs and pubs, which included sports bars, and specific betting facilities. Some participants described how the marketing for sports betting in everyday community spaces created a perception that sports betting was “accepted” and “normal”. Some also specifically described how constant exposure to marketing for these products was so common that they had become “desensitised” to it, and the more they implicitly accepted (or did not question) the presence of gambling marketing in these spaces, “the more you see it the more you think that’s okay”. Others stated that the constant exposure to marketing for sports betting products also removed the stigma traditionally associated with betting by creating a perception that gambling on sports was a normal activity:

I think subconsciously people start thinking it’s okay because they see it all the time…you start seeing it again and again and you think that’s the norm. –26 years., PGSI 1.

**Table 2:** Reported marketing channels for sports betting

Marketing channels (non-gambling environments)	*N* (%)
TV (during sports broadcasts, Footy Show)	50 (100%)
Online (pop-up banners)	25 (50%)
Social media (Facebook, Twitter, YouTube)	18 (36%)
Radio	15 (30%)
Sports stadiums/infield	11 (22%)
Newspaper	11 (22%)
Billboards	10 (20%)
Team Jerseys	7 (14%)
Magazines	2 (4%)
Public transport	2 (4%)
Marketing channels (gambling environments)	*N* (%)
Pub/sports bar	11 (22%)
Mobile (push notifications/apps)	8 (16%)
TAB	4 (8%)
RSL/club	3 (6%)

A key subtheme within participants’ narratives related to the saturation of advertising for sports betting within sporting environments. Participants used words and phrases such as “constant”, “over saturated”, and “it’s absolutely everywhere, it’s impossible to miss” to describe the push of marketing for sports betting into sports. The perception that it was almost impossible to avoid marketing for gambling was a recurring theme throughout participants’ narratives, with many of the narratives implying that some young men felt trapped by the amount of marketing for sports betting products. For example, one participant stated that as a sports fan “you can’t escape it”, with another stating that “it’s the single most marketed thing out there”. Participants described the impact of saturated marketing on the normalisation of betting in sports. Some described this in more general and generic terms stating that because betting advertisements were “in everything now, it just seems normal”. However, others, including some at higher risk levels of gambling harm, described the impact of constantly seeing promotions for inducements and incentives to gamble. The following moderate risk gambler directly attributed the embedding of gambling within sporting cultures to the constant push of gambling products and services throughout sporting matches:

That’s the reason it’s become so dominant in our culture, because it’s so overly pushed in everything that we see and we do to do with sport. Just coming down to the commercial breaks, the half time breaks, it’s focused around betting companies, and what you can bet on. –25 years., PGSI 4.

Similarly, the following participant described the familiarity of seeing very specific promotions for sports betting products when viewing sports events. In particular, he described the specific informational campaigns run by betting companies which were designed to teach individuals about the range of different ways to engage in betting via mobile technologies:

There’s just so much. Particularly in ad breaks, where they’re just jam filled with ads for new odds, ways you can increase your bet, ways that you can change, tap out. All that stuff is in the marketing. –27 years., PGSI 4.

### A symbolic alignment with sports: the role of sponsorship- and commentary-based marketing

Participants described the role of sponsorship deals between industry and sporting codes as creating a symbolic alignment between gambling and sports. In particular, participants described the reliance of two of Australia’s major sporting codes—the AFL and NRL—on gambling revenue via sponsorship relationships. Some commented that this made gambling “even more integrated” into matches, with sporting codes and teams playing an active role in the promotion of betting. Several participants described the way in which implicit endorsement of betting by teams and codes contributed to the normalisation of gambling:

Every team is sponsored by a gambling agency. It’s on every ad break on TV. I mostly watch the NRL and it’s very much promoted by the NRL. – 29 years., PGSI 2.

Participants described the subtle ways in which marketing for sports betting had also become embedded within sports-based commentary before and during sporting matches, as well as in sports-based entertainment shows. For example, some participants described how commentators spoke about the performance of players and teams through an “odds lens”. Others stated that betting “language” was now included in the general sports commentary surrounding the match, with commentary during breaks in play overwhelmingly focused on gambling—“at half time they don’t talk about the game, they just talk about the odds”.

Others described how broadcasters would cross to a bookmaker for the odds of the game—not only on the match outcome but also on individual players and specific statistics associated with the game. Some stated that this changed the terminology and discourse associated with the match, with bookmakers and sports commentators encouraging fans to view the match through a gambling lens:

You watch a game of footy and they generally will cross to the [bookmakers] and ‘these are the odds’. When they bring up the teams on Fox Footy, they say ‘well it’s the Crows and the Power’. And how they determine the favourite isn’t by saying ‘these guys are the favourites’, they show the odds. –24 years., PGSI 3.

Most participants stated that crossing to bookmakers throughout the match had become such a normal part of the game that they rarely thought to challenge the presence of these forms of promotions:

You have your commentators reviewing games and they talk about the odds and who is favourite to the extent whereby they sometimes cross over to someone who gives you the odds on who is trending well. I think it becomes so in your face that we just accept it as normal now. –24 years., PGSI 3.

The “integration” of odds promotions outside of live broadcasts and into sporting programmes was also identified by several participants. In particular, evening panel discussion shows relating to the AFL and the NRL were now considered a platform for the promotion of betting: “when you watch the footy show for the NRL, every time they preview a team for the weekend coming up, the thing that they talk about is the odds”. Some participants perceived that the integration between gambling companies and sporting codes conveyed a message that gambling was an integral part of sports fans’ identities and a precondition to enjoying the match and supporting their team. For example, one participant described how watching sports and following a team was no longer solely about emotion and passion but was also about backing their team through betting:

They’re trying to create an atmosphere where it’s no longer good enough to just passionately follow your team. Not only [do you] back them with your emotions, you back them financially. –29 years., PGSI 0.

### Targeted and vulnerable: the role of marketing in shaping the gambling identities of young men

The majority of participants believed that young men were the key target market for gambling companies and that marketing had played an important role in shaping the gambling identities of young men. Many participants also believed that young men were especially vulnerable to gambling harm, with some perceiving that marketing amplified the risks associated with sports betting: “the marketing targets young men, I think that’s why they’re most at risk”. Participants described a range of marketing appeal strategies that they believed were particularly influential in shaping gambling as a part of sports fan behaviour. The most common was the use of appeals and imagery that centred upon peer belonging and mateship. Participants described advertising creatives which linked betting as something that “you and your mates” participated in. A number of participants spoke about how advertisements sought to align what they already valued and appreciated in sports, with that of betting. For example, the following participant described how effective he thought sports betting marketing strategies were in linking gambling to friendship and comradery.

I think more so than a lot of other advertising, sports gambling advertising really is holding that mirror up to what is good and fun about sport, which is friendship, and comradery, and hanging with your mates, and having a punt while you’re doing that. I think it’s that connection that they are looking for between mateship and having a bet. –29 years., PGSI 0.

Some participants related to the symbolism within sports betting marketing, with one participant stating that the creatives and appeal strategies used within gambling advertising were a “mirror image of the clients they are trying to get”. Some participants stated that they could relate and identify their own relationships and betting behaviours to those portrayed in advertisements:

They make you identify with yourself and your mates as well, and they’re very relatable. They put you in a position of ‘yeah that’s actually happened to me’, it attracts young men definitely. –32 years., PGSI 2.

Others commented that sports betting advertising incorporated existing rituals associated with young men’s sports fan behaviours (e.g. watching sports with peers at a pub) and sought to add gambling to these behaviours. The following comment suggests that these marketing tactics created a strong positive cultural narrative for young men about the role of gambling in their peer groups and their collective identities surrounding sport:

They make it look like it’s a boys club, as if it’s part of what you should be doing, watching the footy and betting, watching the races and gambling, and showing off because you’ve won. There’s that aspect which a lot of the ads are doing now. They’re targeting those younger boys. Like you can be the cool guy that won money and this is what you should be doing - you should be gambling, you should be doing this. –30 year., PGSI 5.

Despite their engagement in betting, some participants remained negative about how embedded gambling marketing had become within sports, with some using words such as “overbearing” and “ridiculous”. Participants argued that it was extremely problematic that individuals could not watch sports without being exposed to the marketing for sports betting and felt frustrated that sports broadcasts were overloaded with betting promotions. A few described switching off the television when they felt too pressured to gamble because of betting promotions:

They’ll be games where I probably won’t bet because I’ve seen the ads and I just get annoyed with it. It’s like, no it’s too much saturating, and it’s almost like pressure. –31 year., PGSI 3.

### Reducing risk and creating feelings of control: the role of inducements and incentives in nudging consumption

Many (*n* = 34) participants considered that the incentives offered by the betting industry were amongst the most effective marketing strategies in getting themselves and others to bet on sports. Described as “the big ones”, inducements and incentives such as “cash back offers” and “bonus bets” were considered to be “safety nets” and softened participants’ perception about the level of risk associated with gambling. Most participants who took up these offers believed that they provided benefit to the consumer, with some perceiving that it would be “silly” not to take advantage of these offers:

I see it as an insurance type of bet really, it’s like if you’re flipping a coin three times, but if you get it wrong twice you get your money back. If you actually analyse it, the value is there. I consider it a smart and reasonably safe, more safe bet. –29 years., PGSI 6.

Participants also believed that they could maximise their winnings through large sign-up bonus bets. Many conceptualised these incentives as free money, with these types of inducements being the most influential mechanism in stimulating the opening of betting accounts. Many described that while they initially thought they would open accounts, use the bets, and then walk away, this was the first step into long-term patterns of gambling:

When all the bookies started to promote the ‘sign up with us with twenty dollars and we’ll give you a hundred dollar bonus bet’, that was enough for me to go ‘oh yeah, fuck it, I’ll just have one big bet, and use their special, their promotional offer. If I win, great, I’ve got some extra money, if I don’t I’ll walk away’. But obviously I never walked away. –34 years., PGSI 3.

Participants noted that inducements led them to open multiple accounts: “I have accounts with everyone, when you sign up you get a bonus bet, so I’ve signed up to all of them”. One participant who was a former problem sports gambler explained that incentives were “dangling carrots” that “hook” you into opening multiple online betting accounts. However, other participants believed that bonus bets and a competitive gambling environment meant that they could take advantage of deals between bookmakers. While several participants considered this to be a risky gambling behaviour—“they give you points, they give you credits, and sometimes they give you money back…that’s risky”—they still actively pursued deals, with many participants ignoring any potential risk—“if they’re giving me $100 bucks to do it, I might as well do it!”. There was evidence that inducements stimulated some participants to continue gambling and to gamble when they otherwise would not have gambled. For example, one participant stated: “I gamble again if they’ve given me something for free”. Even when participants viewed these offers critically, stating that it was important to “see what these companies are doing in the way that they advertise”, this did not always prevent the uptake of betting promotions. For example, even though the following participant knew that inducements were a clever marketing tactic, they still influenced his decisions to place bets and how much he would gamble:

You’re not concerned about the $50 you put on a bet because you’ve got two different ways you can win it back, or potentially get $100. They’re clever in what they do by making things that are actually harmful or addictive and they minimise it to a point where you don’t consider it. –27 years., PGSI 4.

Some specific incentives created a perception amongst participants that they were more in control of their gambling. For example, they described “tap out” promotions which resulted in them believing they could “curb your losses” and “increase your chances of winning”. Others perceived that inducements were generally linked with specific athletes who were “safe” options for betting, for example, those who could perform under pressure or who were regular try or goal scorers. A number of participants stated that these types of inducements were particularly influential in encouraging their decisions to place a bet: “If they say something about a particular player that I like, I will go for it”. Some believed that placing bets on these players gave them a greater degree of control over the outcome of the markets that they gambled on and that incentives would protect their money even if the bet was unsuccessful:

Probably the cash back ones. I had a bet on the Origin [NRL interstate competition], and I had a bet on the first try scorer. I put it on Greg Inglis, knowing that he is more than likely going to score a try during the game, but they give you your money back even if he doesn’t score first, but scores during the game. You get your money back. –34 years., PGSI 2.

Others described the impact of inducements tied to the emotion associated with large sporting events. For example, some explained that inducements and incentives had a significant impact when you were “fired up about watching your team”, with some evidence that they stimulated participants to bet more than they normally would during these events. This was particularly the case for individuals with moderate- or high-risk levels of gambling. For example, the following participant stated that when inducements were offered, it was “easy to have the urge and bet above what you normally do”. Another stated that push notifications via his mobile phone, which offered a range of promotions, led him to bet more than he normally would have done during the State of Origin NRL match:

The Origin a couple of weeks ago is a good example. Most major online companies, they do pretty substantial offers, so if you lose by eight points or less money back, or if you’re losing any time money back. And with those particular games I certainly bet a lot more then what I normally would, based on those specials and promotions. –29 years., PGSI 6.

## Discussion

**Fig. 1:**
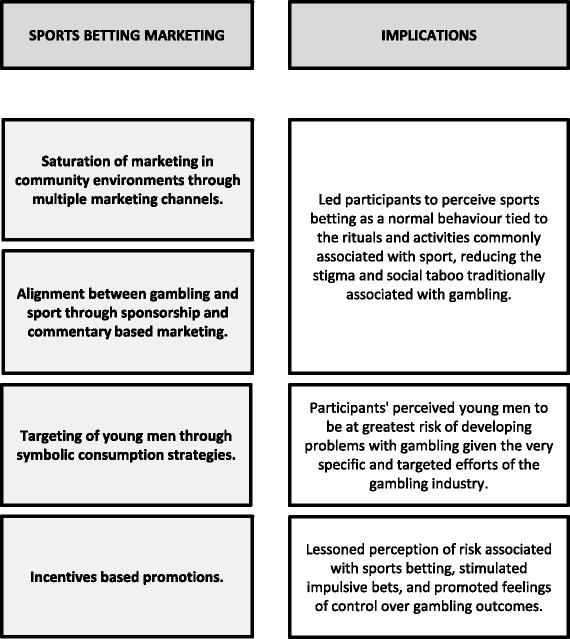
Marketing strategies that may shift the cultural meanings associated with gambling and sports. Legend: Theoretical model of how marketing strategies may influence a shift in the cultural meanings associated with gambling and sports, as well as the distinct promotional factors that may influence gambling consumption behaviours

This study sought to investigate how the marketing of sports betting products may influence betting attitudes and consumption behaviours which ultimately may lead to harm. The study also sought to consider potential strategies to reduce the risks posed by these marketing strategies for young male sports fans. Figure 1 provides a theoretical model of how marketing strategies may influence a shift in the cultural meanings associated with gambling and sports, as well as the distinct promotional factors that may influence gambling consumption behaviours. It is clear from this study that most participants recall the marketing for sports betting in environments which are not specifically designed for gambling, and this was most notably the case during live broadcasts of Australian sports. This is the phase of cultural alignment that McCracken [34] described as taking cultural meanings within the social world, and applying them to products. Marketing for sports betting products is no longer confined to specific gambling environments (such as bookmaker websites or mobile applications and gambling venues). Rather, the marketing for these products has entered everyday community and media spaces, which have not traditionally been aligned with gambling. Participants’ narratives suggest that the embedding of sports betting marketing in sports, termed by McMullen & Miller [11] as the “gamblification” of sports, has created a new cultural meaning that betting is core to the sporting experience. We would argue this is increasingly similar to betting being core to the experience of horse racing.

However, it is not only the placement of sports betting advertisements within sports but the saturated nature of these promotions that has exacerbated a cultural alignment between betting and sports and the subsequent risks associated with betting. Constant saturation marketing, particularly during sporting matches and programmes, influenced participants’ beliefs that gambling had become a normal or common part of sports. This finding is similar to research conducted with children and adolescents reflecting that 75% of young people believed that gambling is a normal or common part of sports, based on the marketing for gambling products during sporting matches [13]. This raises an important issue for policy makers and regulators in relation to the prevention of harm. While marketing for some forms of gambling (e.g. EGMs or “pokies”) is prohibited in Australia, marketing for sports betting continues with limited regulatory frameworks. Recognising the potential for harm, some countries, such as the UK, are now considering the prohibition of gambling promotions before the watershed (the point in time after which programmes with adult content may be broadcast), as part of a comprehensive suite of harm reduction strategies associated with sports betting products [46]. In Australia, the state of Victoria is considering the restriction of betting promotions on public transport and infrastructure near schools [47]. This study suggests that policies aiming to denormalise gambling as an inherent part of sports must consider significant restrictions on the volume of advertising during sports programmes, sporting matches, and within non-gambling environments at any time of the day.

The study indicates that there are specific marketing mechanisms that intensify the symbolic relationship between gambling and sports. We would argue that the endorsement of betting by sporting codes (particularly via sponsorship relationships) and broadcasters has a significant influence in betting becoming a meaningful part of the life of sports fans. In part, this is because endorsement by these agencies contributes to the removal of the negative social stigma once associated with betting, embedding it as a valuable social norm for the fans of sporting codes. Norms are regulated, in part, by the images and narratives created about sports betting by sporting codes and sporting commentary panels (often comprised of sporting heroes). McCracken [36] argues that celebrity endorsement has a powerful impact on products because, as compared to anonymous models, celebrities “deliver meanings of extra subtlety, depth, and power”. We would expand McCracken’s definitions of “celebrity” to sporting codes, which are able to provide powerful positive endorsements of gambling which may in turn (a) lead to harm and (b) help to facilitate the transfer of cultural meanings associated with betting products to the participants themselves. The endorsement by sports codes and commentators appeared to have the strongest influence on encouraging individuals to see sports through a “gambling lens”. Given the reported influence of these types of marketing by young men in our sample, and also from studies with children [13], we would argue that the embedding of commentary-based marketing in sports is clearly an issue that requires urgent consideration by governments and sporting authorities to prevent risk and the potential for harm.

The results also suggest that very specific forms of promotions, such as inducements and incentives, play a significant role in establishing betting on sports as part of consumer behaviour. These strategies must be of central focus in any regulatory efforts to prevent and reduce harm. Inducements and incentives minimised the perceptions of risk associated with sports betting, promoted feelings of control over the betting outcomes, and encouraged individuals to open more accounts with gambling companies and gamble more than they normally would, including on events on which they might not otherwise gamble. Even when participants acknowledged that these types of promotions were a clever marketing tactic that could increase the risks associated with gambling, they still had a strong influence over gambling consumption intentions. Inducements are not a new marketing phenomenon for the gambling industry and are used on a range of different gambling products to stimulate consumption [48]. However, our research contributes to growing evidence about the significant influence that these types of marketing promotions may have in encouraging risky gambling behaviours. While other researchers have shown that advertising may be particularly influential in “impulse” gambling behaviours of problem gamblers [29], our study suggests that these inducements may also be influential in stimulating the gambling consumption patterns of young male sports gamblers with low and moderate levels of gambling problems. Given that research also suggests that promotions for inducements may have high recall amongst young people and may be influencing young people’s perceptions that gambling is a “risk free” activity [13], regulation is urgently needed to prohibit the marketing for these particularly influential types of promotions.

We would also support the development of sustained and adequately funded public education programmes to complement the legislative approaches already suggested for policy makers. These programmes should be developed independent of the gambling industry and related interests, emphasise the harms associated with their products, and expose approaches used by the industry. There is encouraging evidence on the value of mass media campaigns in addressing a range of public health problems [49], and the findings from this study should assist in providing formative research for the development of such campaigns related to gambling. In addition to this, further research should also seek to explore the influence of marketing on the betting attitudes and behaviours of other population subgroups, including female sports fans, and younger populations, such as children and adolescents.

Finally, it is important to consider the study limitations. First, a recruitment criterion for the study was that participants wagered on two of Australia’s major football codes (NRL or AFL). Despite participants having gambled on a range of products, the results of our study may not be generalisable to young men who bet on other sports, horses, or sporting events (e.g. Tennis). Second, the sample was skewed towards young men who were educated and living in more affluent socio-economic neighbourhoods. Finally, in this study, we did not aim to diversify the sample with regard to ethnicity.

## Conclusions

This research suggests that marketing plays a strong role in the normalisation of gambling in sports and in encouraging gambling consumption intentions and behaviours. For the young men in our study, the emotional investment in the game, and the ways in which they 'consume sport', for the most part, now included betting on the match. In aligning gambling with culturally valued entities, and pushing numerous incentivisation strategies through ubiquitous marketing channels, the gambling industry is influencing not only individuals’ gambling risk perception but also the level to which they engage in gambling. This is problematic as there is an absence of overarching cultural and organisational structures to restrict sports betting promotions. There is now a clear industry presence in non-gambling and community settings. Policy makers must begin to consider the lag between evolving gambling landscapes and sophisticated marketing strategies used by the gambling industry (and sporting codes) to promote gambling products, and effective harm reduction measures in order to protect populations from gambling harm.

## Abbreviations

AFL: Australian Football League
CGT: Constructivist grounded theory
EGM: Electronic gambling machines
NRL: National Rugby League
PGSI: Problem Gambling Severity Index
SEIFA: Socio-Economic Index for Areas Score
TAFE: Technical and Further Education
